# Trends in Internal Medicine Faculty by Sex and Race/Ethnicity, 1980-2018

**DOI:** 10.1001/jamanetworkopen.2020.15205

**Published:** 2020-09-01

**Authors:** S. Michelle Ogunwole, Michael Dill, Karen Jones, Sherita H. Golden

**Affiliations:** 1 Department of Medicine, Johns Hopkins University School of Medicine, Baltimore, Maryland; 2 Johns Hopkins Center for Health Equity, Baltimore, Maryland; 3 Association of American Medical Colleges, Washington, DC; 4 Welch Center for Prevention, Epidemiology, and Clinical Research, Baltimore, Maryland; 5 Johns Hopkins Medicine Office of Diversity, Inclusion, and Health Equity, Baltimore, Maryland

## Abstract

**Importance:**

Increasing diversity in the physician workforce is a fulcrum for reducing health disparities. Efforts to increase the diversity in the internal medicine (IM) workforce may improve health equity among an increasingly diverse population with increasing prevalence of chronic disease.

**Objectives:**

To assess diversity trends in the academic IM workforce and evaluate how well these trends reflected medical student diversity and the changing demographic composition of the general population.

**Design, Setting, and Participants:**

This secondary analysis of a cross-sectional study analyzed data from January 1, 1980, to December 31, 2018, from the Association of American Medical Colleges Faculty Roster and Applicant Matriculant File, which capture full-time US medical school faculty and matriculants, respectively, and population data through 2017 from the US Census Bureau.

**Main Outcomes and Measures:**

The study calculated the proportions of women and individuals from racial/ethnic groups that are traditionally underrepresented in medicine (URM) among IM faculty and faculty in all other clinical departments. These data were compared with the proportions of female and URM matriculants in US medical schools and the proportions of women and individuals from underrepresented racial/ethnic groups in the population. The analysis was stratified by sex, race/ethnicity, and intersections of sex and race/ethnicity.

**Results:**

From 1980 to 2018, the absolute number of full-time IM faculty increased from 10 964 to 42 547. Although IM was the department classification with the most women faculty, in 2018 it continued to have a lower proportion of women (n = 17 165 [40.3%]) compared with all other clinical departments (n = 48 936 [43.2%]). Among IM faculty, the percentage of URM faculty members more than doubled during the study period (from 4.1% to 9.7%) but still made up only a small portion of faculty members. The percentage of female matriculants among medical school matriculants increased steadily (from 28.7% in 1980 to 51.6% in 2018) and was nearly identical to their population representation in 2017 (50.7% compared with 50.8%). Although the percentage of URM matriculants had nearly doubled since 1980 (from 11.3% to 18.1%), it still lagged far behind the proportion of individuals in the US population who are members of underrepresented racial/ethnic groups (18.1% vs 31.5% in 2017).

**Conclusions and Relevance:**

This cross-sectional study found that progress has been made in diversifying academic IM faculty; however, it does not yet reflect the diversity of medical students or the US population. Continued efforts to increase the diversity of the academic IM workforce are needed.

## Key Points

**Question:**

Did sex and racial/ethnic diversity in the academic internal medicine workforce between 1980 and 2018 reflect the changing demographic composition of the general population (in 2017) and medical student body?

**Findings:**

This cross-sectional study used data on full-time medical school faculty appointed to US internal medicine departments from 1980 to 2018, matriculants to US medical schools from 1980 to 2018, and the general US population from 1980 to 2017. The study found that progress was made in diversifying the academic workforce; however, it may not yet reflect the diversity of US medical students or the US population.

**Meaning:**

These findings suggest that continued improvement of recruitment and retention efforts for women and groups who are underrepresented in medicine as medical students and faculty in internal medicine is needed.

## Teaser

This secondary analysis of a cross-sectional study of internal medicine faculty and medical school matriculants assesses diversity trends in the academic internal medicine workforce and evaluates how well these trends reflect medical student diversity and the changing demographic composition of the general population.

## Introduction

By 2060 the US population will have undergone significant demographic changes. The US Census Bureau projects that between 2014 and 2020 the population will have increased by nearly 100 million people, reaching 417 million, and the number of adults 65 years and older is expected to increase by nearly 75% between 2020 and 2060 (from 56.4 million to 98.2 million).^1^ The racial and ethnic makeup of the US population will change as it becomes a minority-majority nation, with racial/ethnic minorities constituting 56.4% of the population by 2060.^1^ Another anticipated change is an increase in the number of adults with multiple chronic medical conditions,^2,3^ and patients with multiple chronic conditions use more health services than other individuals and account for approximately 83% of all health care spending.^4,5^

Taken together, these projected changes will bear directly on the health and economy of future generations, and a larger, more diverse physician workforce focused on the prevention and management of complex chronic disease (such as internal medicine [IM] and its subspecialties) could help meet the nation’s increasing health care needs and lead to improved patient outcomes.^6,7,8,9^

Because IM faculty serve as teachers and direct role models for medical students, they play an instrumental role in recruitment into this field. Indeed, diversity among faculty is associated with diversity and cultural competence among students and a reduction of health-related disparities.^10,11,12,13^ However, it is unclear to what extent diversity among IM faculty reflects the diversity of the nation’s population or the diversity of the medical student body. Our aim in conducting this study was to add to existing research^14,15,16^ on demographic trends among faculty in other specialties by analyzing the sex and race/ethnicity composition of IM faculty, other medical school faculty, medical school matriculants, and the general US population. We also examined these trends through the intersection of sex and race/ethnicity.

## Methods

This secondary analysis of a cross-sectional study used data on full-time medical school faculty appointed to IM departments in the US from January 1, 1980, to December 31, 2018, matriculants to US medical schools that granted doctor of medicine (MD) degrees from January 1, 1980, to December 31, 2018, and the general US population from January 1, 1980, to December 31, 2017 (the most recent data available) to describe and compare sex- and race/ethnicity-specific trends in these groups over time. We obtained medical school faculty data from the Association of American Medical Colleges (AAMC) Faculty Roster, a comprehensive national database of full-time US medical school faculty.^17^ Faculty were classified based on department, not medical specialties; thus, IM faculty included general IM and other subspecialties. We obtained medical school matriculant data from the AAMC Applicant Matriculant File.^18^ We obtained general population data from the US Census Bureau national population data sets.^19,20,21,22^ For US Census Bureau and medical student data from the AAMC Applicant Matriculant File, race/ethnicity and sex are self-reported; however, faculty race/ethnicity and sex data from the AAMC Faculty Roster are not considered self-reported because they are reported to the Faculty Roster by the faculties’ institutions and then supplemented by other AAMC data sources. Additional information regarding the reporting and classification of race/ethnicity can be found in the eAppendix in the Supplement. This secondary analysis used deidentified data; however, in the original data collection, data were identifiable, and written informed consent was obtained. This secondary analysis was deemed exempt from guidelines for research involving human participants by the institutional review board of the American Institutes for Research.

### Statistical Analysis

We calculated the proportions of women and individuals from racial/ethnic groups who are underrepresented in medicine (URM) for IM faculty and faculty in all other clinical departments. Underrepresented in medicine is defined as Hispanic (Hispanic, Latino, or of Spanish origin in combination with any other race or ethnicity), Black (Black or African American, alone only), Native Hawaiian or other Pacific Islander (alone only), and American Indian or Alaska Native (alone only). We then compared these proportions with the proportion of female and URM matriculants in US MD-granting medical schools and the proportion of women and individuals from traditionally underrepresented groups in the general population. To allow for comparability, we defined *underrepresented minorities* as the same groups who compose URM faculty and matriculants (ie, Hispanic, Black, Native Hawaiian or other Pacific Islander, and American Indian or Alaska Native).

The χ^2^ test was used to compare IM faculty with all other clinical faculty in 2018. To assess trends in sex and URM status between 1980 and 2018, we estimated the slope and associated probability values for each group by using simple linear regression models in which year was used as an independent variable. A 2-sided *P* < .05 was considered to be statistically significant. Statistical analyses were conducted using SAS software, version 9.4 (SAS Institute Inc).

## Results

From 1980 to 2018 the absolute number of full-time IM faculty increased almost 4-fold (from 10 964 to 42 547) (Table), and IM retained its broader status as the clinical practice specialty with the highest number of physicians during those years.^17^

**Table.** Internal Medicine Faculty, Faculty in Other Clinical Departments, and Medical School Matriculants, 1980 and 2018^a^

Group	Total No.	No. (%)							
		Sex			URM status			Female^b^	
		Male	Female	Undetermined	URM	Not URM	Undetermined	URM	Not URM
Internal medicine faculty									
1980	10 964	9804 (89.4)	1160 (11.8)	0	453 (4.1)	10 016 (91.4)	495 (4.5)	62 (13.7)	1049 (10.5)
2018	42 547	25 382 (59.7)	17 165 (40.3)	0	4128 (9.7)	35 720 (84.0)	2699 (6.3)	1861 (45.1)	14 092 (39.5)
Other clinical faculty									
1980	29 812	24 488 (82.1)	5324 (17.9)	0	1468 (4.9)	26 960 (90.4)	1384 (4.6)	361 (24.6)	4704 (17.4)
2018	113 365	64 429 (56.8)	48 936 (43.2)	0	11 065 (9.8)	94 795 (83.6)	7505 (6.6)	5672 (51.3)	39 296 (41.5)
Matriculants									
1980	16 587	11 830 (71.3)	4757 (28.7)	0	1869 (11.3)	14 563 (87.8)	155 (0.9)	714 (38.2)	4003 (27.5)
2018	21 622	10 454 (48.3)	11 160 (51.6)	8	3921 (18.1)	15 570 (72.0)	2131 (9.9)	2132 (54.4)	7933 (51.0)

Abbreviation: URM, underrepresented in medicine.

^a^ Data are from the Association of American Medical Colleges Faculty Roster as of June 27, 2019, and the Association of American Medical Colleges Applicant Matriculant File as of May 20, 2019. URM is defined as Hispanic (regardless of other identities), Black only, American Indian or Alaska Native only, or Native Hawaiian or other Pacific Islander only. Non-URM is defined as White only or Asian only.

^b^ This column shows the intersection of (female) sex and URM status.

### Comparison by Sex

**Figure 1.**
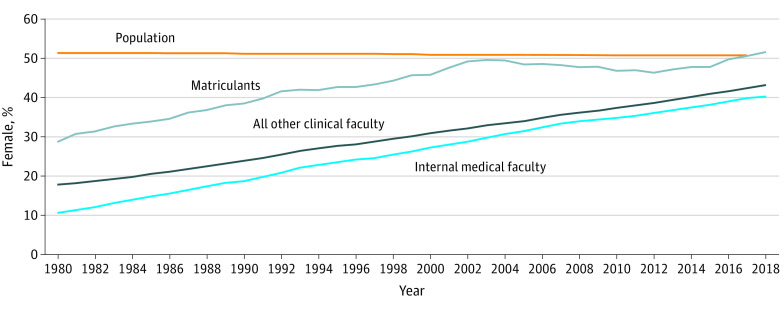
Percentage of Internal Medicine Faculty, Faculty in Other Clinical Departments, Medical School Matriculants, and the US Population Who Were Female, 1980-2018

Data sources were limited to only 2 categories for sex, female and male. When we looked at changes in the percentage of women over time, we found that all trends (slopes) were positive and statistically significant (slopes of 0.79 for IM faculty, 0.67 for other clinical faculty, and 0.52 for matriculants; *P* < .001 for all). The proportion of women among the IM faculty increased steadily but still remained 10.9% lower than their representation in the US population in 2017 (39.9% vs 50.8%) (Figure 1). Even though IM was the department with the largest number of female faculty, it continued to have a lower proportion of women in 2018 when compared with all other clinical departments (40.3% vs 43.2%; χ^2^ = 100.9; *P* < .001). On the other hand, women made appreciable gains in representation among entering medical students. The data indicate a steady increase in female representation among matriculants to 2004, followed by a period of decline but increasing to a peak of 51.6% in 2018, exceeding the 2017 population mean (Figure 1).

### Comparison by URM Status

The prevalence of URM individuals among faculty and medical students increased, although not to the same extent as female representation. When changes in the percentage of URM individuals over time were examined, all trends (slopes) were positive and statistically significant (slopes of 0.17 for IM faculty, 0.13 for other clinical faculty, and 0.14 for matriculants; *P* < .001 for all).

**Figure 2.**
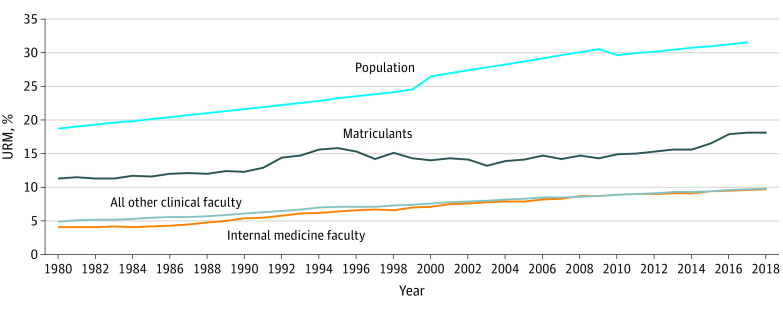
Percentage of Internal Medicine Faculty, Faculty in Other Clinical Departments, Medical School Matriculants, and the US Population Who Were Underrepresented in Medicine (URM), 1980-2018

For IM faculty, the percentage of URM individuals has more than doubled during the past 38 years (4.1% vs 9.7%) (Figure 2). However, in 2018, URM groups still made up a small portion of IM faculty, which was nearly identical to the URM representation in all other clinical departments (9.7% vs 9.8%, χ^2^ = 0.120, *P* = .73). For matriculants, the URM percentage increased in the early 1990s, reaching an initial peak of 15.8% in 1995 before declining until 2003 and then increasing again to an all-time high of 18.1% in 2018.^17^ Although the percentage of underrepresented minorities in the US population increased from 18.7% to 31.5% between 1980 and 2017, these changes were not reflected in medical education. In 2018, the proportions of URM groups in IM (9.7%), in other clinical departments (9.8%), and among medical school matriculants (18.1%) remained well below the proportion of underrepresented minorities in the general population (Figure 2).

### Comparison of the Intersection of Sex and URM Status

**Figure 3.**
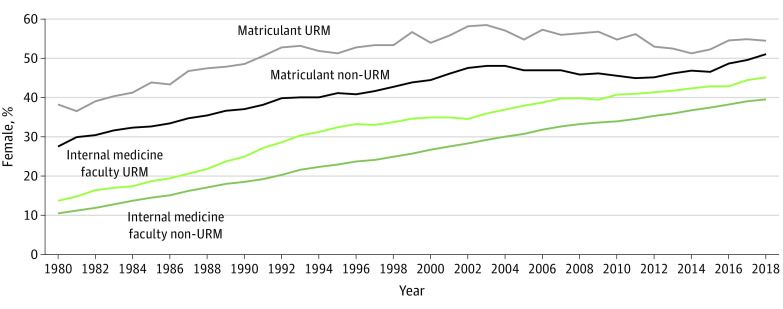
Percentage of Underrepresented in Medicine (URM) and Non-URM Internal Medicine Faculty and Matriculants Who Were Female, 1980-2018

As Figure 3 shows, consistent with the broader trend among medical school faculty, female representation among URM groups as a percentage of IM faculty increased, and female faculty had greater representation among URM faculty than they did among non-URM faculty. However, the absolute number of URM IM female faculty was small compared with the non-URM female IM faculty (1861 vs 14 092 in 2018). Among medical school matriculants there was a similar pattern: higher female representation among the URM matriculants compared with the non-URM matriculants and a smaller absolute number of URM female matriculants compared with the non-URM female matriculants (2132 vs 7933 in 2018).

**Figure 4.**
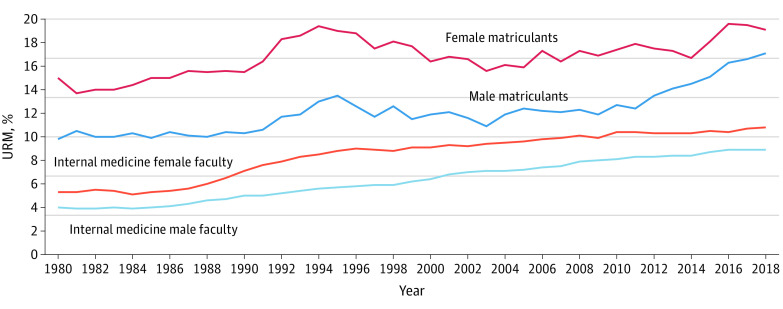
Percentage of Underrepresented in Medicine (URM) Internal Medicine Faculty and Medical School Matriculants by Sex, 1980-2018

Comparing the percentages of URM female and male IM faculty, starting in the late 1980s and continuing through the 1990s, URM proportions among female IM faculty appeared to increase faster than those among male IM faculty, but beginning around 1996, the increase in the percentage of URM female faculty slowed, whereas growth remained steadier among male faculty (Figure 4). The percentage of female IM faculty who were URM remained at least 1 percentage point above that for male IM faculty who were URM, a relationship that remained for the almost 30 years of the study period (1.3% higher in 1980 and 1.9% higher in 2018) (Figure 4).

Among URM female and male matriculants, from 1980 to 1994 there were also notable trends. There was a sharp increase in URM female medical school matriculants that peaked at 19.4%. This number decreased after 1994, and then in 2006 the proportion began to increase again, reaching 19.1% in 2018. The percentage of URM male matriculants decreased slightly after 1994 but increased to 17.1% by 2018.

## Discussion

In this cross-sectional study, despite improved representation, women, URM individuals, and perhaps most especially those who share these dual identities, continued to face challenges in terms of true representation in academic medicine. According to current literature, these challenges are associated with a lack of parity in compensation,^23,24^ promotion, leadership opportunities,^25^ and, at their worst, frank discrimination and harassment.^26,27,28,29^ Equally concerning are the increased levels of isolation, burnout, and attrition in academic medicine.^30^

These factors have been associated with recruitment and retention in academic medicine and may have implications for disparities in patient outcomes. Specifically, patient-physician concordance in race, ethnicity, and language has been associated with improved access to care, health outcomes, and patient satisfaction in minority patients.^12,31^ Similarly, patient-physician sex concordance is associated with greater preventative services use and satisfaction among female patients.^32,33^ Consequently, a dearth of diversity in the IM physician workforce may hamper efforts to reduce health-related disparities for the rapidly diversifying US population. Moreover, just as greater faculty diversity has been associated with greater student diversity and richer experiences for URM students,^34,35,36^ greater diversity among IM faculty may help create a more diverse IM workforce for the future.

This study of trends in the composition of IM and other clinical faculty by sex and race/ethnicity suggests that diversity among IM faculty and medical school matriculants is increasing. Although female medical school matriculants approached a level of representation reflective of the population, this was not the case for female faculty in IM or female clinical faculty in general, although substantial gains were seen during the study period. For URM groups, neither IM clinical faculty nor medical school matriculants approached population percentages, and trends do not suggest that they will. In part, this may be because of the challenges to affirmative action that began around 1995^35,37,38^; for example, between 1990 and 1994, the Project 3000 by 2000 campaign launched by the AAMC successfully helped to increase the number of URM matriculants by 36%.^37^ The present study saw a corresponding peak in the percentage of URM matriculants during this time; however, the campaign was ultimately unsuccessful in meeting its goals, likely hindered, in part, by state ballot initiatives to ban affirmative action. In subsequent years, our study found, there was a decrease in the percentage of URM matriculants.

Although legislative challenges related to affirmative action may have played a role, the disparities seen in this study are too stark to attribute them solely to those conflicts. Possible causes of the differences observed in the percentages of IM clinical faculty and medical school matriculants who are URM warrant further investigation. Prior work^39^ found that although URM minority matriculation was increasing for male and female Hispanic students and Black female students, Black males were faring less well. This finding may explain why the present study showed overall trends toward increases in the percentages of female matriculants and IM faculty who were URM but less pronounced trends for URM men. The data in the present study suggest that although progress has been made, a need to focus recruitment efforts in academic IM remains .

A lack of diverse role models among IM faculty also may amplify the difficulties associated with increasing IM faculty diversity. Almost one-third of the US population are underrepresented minorities, whereas only 18.1% of matriculants and 9.7% of clinical IM faculty in this study were individuals from URM groups. A lack of representation of women and URM individuals in academic IM may contribute to a medical student body unable to see a future in academic IM.^40^ As an increasingly diverse medical student population moves through the pipeline and completes training, it remains to be seen how many will move into faculty positions.

Renewed attention and innovation around diversity and inclusion are needed to improve representation and retention of women and underrepresented groups in academic IM.^35,41,42^ Efforts in line with this mission include incentives to reduce loan burdens,^7^ changing traditional promotion criteria to include activities to which women and URM individuals may disproportionately contribute (eg, cultural competence curriculum development, diversity committee representation, and social science research),^43^ hiring and promoting diverse faculty to serve as mentors to medical students and junior faculty^44,45^ (without unduly burdening them with the sole responsibility for mentoring female and URM students), reinvigorating the pipeline to medical school through sustained community outreach to ensure access to resources and opportunities to attend and succeed in medical school,^46^ supporting race-conscious admissions policies and challenging anti–affirmative action legislature that can hamper efforts toward sustained underrepresented minority recruitment,^37^ experimenting with technology and other practices that can enhance job flexibility,^36^ and getting organizations to think of innovative ways to promote general IM from a national level.^7^ However, the most effective evidence-based strategies appear to be those focused on shifting culture, which requires change that is supported by leadership and paired with accountability mechanisms.^41,47,48,49^

The nation needs physicians who understand the importance of disease prevention, excel at chronic disease management, and routinely integrate social and economic determinants of health into the practice of medicine.^50^ Internal medicine physicians represent the largest specialty in medicine, and they focus on the comprehensive care of adults with complex medical problems, making them ideally suited to play a significant role in managing the challenges that lie ahead.^7^ Therefore, a special emphasis must be placed on increasing diversity among IM faculty to avoid perpetuating disparities in the field and in the health of the population.^51^

### Limitations

This study has limitations. First, there were variations in the way that race/ethnicity was reported (self-report vs institutional report; see the Methods) and classified. The classification of race/ethnicity changed in the US Census Bureau between 1990 and 2000. The designation Native Hawaiian or Other Pacific Islander had been included in the Asian and Pacific Islander race/ethnicity group but was eventually reported separately. As a result, the Native Hawaiian or Other Pacific Islander population is not included in the underrepresented minority population counts before 2000. In addition, the US Census Bureau’s data only show a true population count every 10 years; interim years show the intercensal data, which are extrapolations and not true counts. The inclusion of only the alone categories for Black, Native Hawaiian, and Native American groups may undercount the URM population for later years when individuals could select multiple race categories. Second, we cannot confirm that sex was self-reported in the AAMC Faculty Roster. Furthermore, we recognize that the reporting of sex does not capture the intricacies of gender identity, which plays an important role in targeting diversity and inclusion efforts. Also, with faculty data, the comparisons made in this study are based on faculty department and not specialty; for IM, this approach does not allow for the identification of the actual practice specialties of faculty members.^20^ Although we did not have these data available at the faculty level, 2 recently published articles^52,53^ examined trends by sex and race/ethnicity for IM residents and IM subspecialty fellows. Santhosh and Babik^52^ found that although the overall total of IM residents and IM subspecialty fellows increased from 2006 to 2018 (from 21 855 to 26 228 residents and 8144 to 10 578 fellows), the proportion of URM IM residents was unchanged (2688 [12.3%; 95% CI, 11.9%-12.7%] to 3599 [13.7%; 95% CI, 13.3%-14.1%]; *P* = .28) but increased to various extents for all subspecialty fellowships. Stone et al^53^ examined trends related to sex and found that from 1991 to 2016 the absolute number and the proportion of female IM residents increased substantially (5602 [30.2%] to 10223 [43.2%]), but the proportion of female IM subspecialty fellows decreased during this same period (33.3% in 1991 and 23.6% in 2016). In addition, given the cross-sectional nature of these data and a lack of information regarding faculty and student experiences, we cannot infer causality from any of the results presented.

## Conclusions

This cross-sectional study found that although progress was made in diversifying the demographic makeup of IM clinical faculty from 1980 to 2018, it may not yet reflect the diversity of the current population or of medical school matriculants. As the health care system is faced with caring for an increasingly diverse, aging, chronic disease–laden patient population, the urgency to meet the needs of patients is escalating, and the importance of securing a diverse physician workforce that can best serve them is crucial. Internal medicine continues to be the largest specialty, and internists have a broad effect on health care, playing a key role in providing primary and subspecialty care.^7^ Continued improvement of recruitment and retention efforts for female and URM medical students and faculty in IM has the potential to significantly increase the size and diversity of the IM physician workforce and improve the quality of, and access to, comprehensive and equitable care.

## Notes

Supplement. **eAppendix.** Association of American Medical Colleges (AAMC) Faculty Roster and Applicant Matriculant File Definitions and Limitations
