# Supplementary material for: Trends in Internal Medicine Faculty by Sex and Race/Ethnicity, 1980-2018
Source: JAMA Netw Open. 2020 Sep 1;3(9):e2015205. doi: 10.1001/jamanetworkopen.2020.15205 (PMC7489836; doi:10.1001/jamanetworkopen.2020.15205)
Supplement: Supplement. — eAppendix. Association of American Medical Colleges (AAMC) Faculty Roster and Applicant Matriculant File Definitions and Limitations [file jamanetwopen-e2015205-s001.pdf]

Supplementary Online Content

Ogunwole SM, Dill M, Jones K, Golden SH. Trends in internal medicine faculty by sex and race/ethnicity, 1980-2018. *JAMA Netw Open*. 2020;3(9):e2015205. doi:10.1001/jamanetworkopen.2020.15205

eAppendix. Association of American Medical Colleges (AAMC) Faculty Roster and Applicant Matriculant File Definitions and Limitations

This supplementary material has been provided by the authors to give readers additional information about their work.

© 2020 Ogunwole SM et al. *JAMA Network Open.*

eAppendix. Association of American Medical Colleges (AAMC) Faculty Roster and Applicant Matriculant File Definitions and Limitations

Association of American Medical Colleges Faculty Roster: Key Definitions and Limitations

Introduction: Medical School Faculty data obtained from the AAMC Faculty Roster are frequently used by researchers. There are important definitions and limitations that should be publicly available to aide researchers in understanding how to best utilize this database. Here we present important definitions and limitations related to the data housed in this data file.

Key Definitions:

AAMC Faculty Roster: a comprehensive national database of full-time U.S. medical school faculty

Faculty Administrative Management Online User System (FAMOUS): an online collection created by the AAMC that is representative of the AAMC Faculty Roster. FAMOUS data is continuously changing and researchers who capture data using FAMOUS must reference the date of data capture.

Underrepresented in medicine (URiM): Hispanic, Latino, or of Spanish Origin (in combination with any other race or ethnicity), Black or African American (alone only), Native Hawaiian or Other Pacific Islander (alone only), and American Indian or Alaska Native (alone only)

Information about the database:

The data pulled from FAMOUS show counts of full-time faculty by department classification. The Faculty Roster utilizes the official name given to each department by each medical school. The department classification is a way to group disparately named (but similar) departments across medical schools. As an example, a medical school could have separate departments of Neurosurgery and Cardiothoracic Surgery. These departments would appear in the Faculty Roster under their official names, but both would be reported in the Surgery department classification.

Limitations:

A. It is important to note that there is a distinction to be made between department classification and specialty. Faculty may hold an appointment in a department that is different from what their specialty is in. For example, someone with a specialty in psychiatry might hold an appointment in a department classified as Internal Medicine. In the Faculty Roster, this psychiatrist would be counted as Internal Medicine faculty.
B. URiM may be undercounted in the Faculty Roster because non-Hispanic faculty with multiple races/ethnicities are grouped together in a single break out called “Multiple Race – Non-Hispanic”. For example, data on faculty who self-report as Black and/or American Indian only includes those faculty who are reported as *only* Black or *only* American Indian and any faculty member who is reported as Black *and* American Indian is thus not counted as URiM in the file.

Association of American Medical Colleges Applicant Matriculant File:

© 2020 Ogunwole SM et al. *JAMA Network Open.*

Key Definitions and Limitations

Introduction:

Medical School Matriculant data obtained from AAMC Applicant Matriculant file is an important data source for many researchers. There are many nuances in the collection and reporting of race/ethnicity data within the file. Over time, changing definitions of race/ethnicity classifications as well as changes in the process of data collection have occurred. Though important to fully disclose these changes, it may not be feasible to include all this information in a manuscript. Here we present important definitions and limitations related to race/ethnicity data in the AAMC Applicant Matriculant file.

Key Definitions:

"Alone" race/ethnicity counts refer to displaying race/ethnicity responses for only those who selected one race/ethnicity. Those who selected more than one race/ethnicity would then be grouped into the "Multiple Race/Ethnicity" category.

"Alone and In Combination" race/ethnicity counts refer to displaying race/ethnicity responses for those who selected one race/ethnicity *and* those who selected more than one race/ethnicity response. For this type of count, there would be no "Multiple Race/Ethnicity" category. Instead, those who selected more than one race/ethnicity would be represented in more than one category.

Underrepresented in medicine (URiM) : Hispanic, Latino, or of Spanish Origin (in combination with any other race or ethnicity), Black or African American (alone only), Native Hawaiian or Other Pacific Islander (alone only), and American Indian or Alaska Native (alone only)

Changes in collection of data:

A. Prior to 2002-2003, applicants could select only one race/ethnicity response. Therefore, the data prior to 2002-2003 represents race/ethnicity counts alone, not in combination with any other race/ethnicity.
B. From 2002-2003 until 2012-2013, the methodology for acquiring race/ethnicity was updated to one question asking an applicant's Hispanic origin and a second question asking an applicant's race.
C. Starting in 2013-2014, the methodology for acquiring race/ethnicity information was updated again. Rather than one question asking an applicant's Hispanic origin and a second question asking an applicant's race, the Hispanic origin and race categories are now listed together under one question about how applicants self-identify. This question allows multiple responses. Additionally, a new response option, "Other", was added. The change in methodology means that data collected from 1980-1981 through 2016-2017 may not be directly comparable.
D. During the 2016 AMCAS application cycle, a technical malfunction in the collection of race/ethnicity data necessitated a request that applicants review and re-submit responses to the race/ethnicity question in their AMCAS applications. Applicants in previous years were not asked to review responses to this question.

© 2020 Ogunwole SM et al. *JAMA Network Open.*
